# Effects of Murine and Human Bone Marrow-Derived Mesenchymal Stem Cells on Cuprizone Induced Demyelination

**DOI:** 10.1371/journal.pone.0069795

**Published:** 2013-07-26

**Authors:** Jasmin Nessler, Karelle Bénardais, Viktoria Gudi, Andrea Hoffmann, Laura Salinas Tejedor, Stefanie Janßen, Chittappen Kandiyil Prajeeth, Wolfgang Baumgärtner, Annemieke Kavelaars, Cobi J. Heijnen, Cindy van Velthoven, Florian Hansmann, Thomas Skripuletz, Martin Stangel

**Affiliations:** 1 Department of Neurology, Hannover Medical School, Hannover, Germany; 2 Center for Systems Neuroscience, Hannover, Germany; 3 Department of Trauma Surgery, Hannover Medical School, Hannover, Germany; 4 Department of Pathology, University of Veterinary Medicine Hannover, Hannover, Germany; 5 Department of Symptom Research, University of Texas, M.D. Anderson Cancer Center, Houston, Texas, United States of America; 6 Laboratory for Neuroimmunology and Developmental Origins of Disease, University Medical Center Utrecht, Utrecht, The Netherlands; University of Muenster, Germany

## Abstract

For the treatment of patients with multiple sclerosis there are no regenerative approaches to enhance remyelination. Mesenchymal stem cells (MSC) have been proposed to exert such regenerative functions. Intravenous administration of human MSC reduced the clinical severity of experimental autoimmune encephalomyelitis (EAE), an animal model mimicking some aspects of multiple sclerosis. However, it is not clear if this effect was achieved by systemic immunomodulation or if there is an active neuroregeneration in the central nervous system (CNS). In order to investigate remyelination and regeneration in the CNS we analysed the effects of intravenously and intranasally applied murine and human bone marrow-derived MSC on cuprizone induced demyelination, a toxic animal model which allows analysis of remyelination without the influence of the peripheral immune system. In contrast to EAE no effects of MSC on de- and remyelination and glial cell reactions were found. In addition, neither murine nor human MSC entered the lesions in the CNS in this toxic model. In conclusion, MSC are not directed into CNS lesions in the cuprizone model where the blood-brain-barrier is intact and thus cannot provide support for regenerative processes.

## Introduction

Multiple sclerosis (MS) is a chronic inflammatory disease of the central nervous system (CNS) that affects mostly young adults [1]. It leads to focal inflammatory demyelination, gliosis, and axonal damage. Remyelination is the natural repair mechanism of demyelination and it was proposed that remyelination might protect from axonal loss and thus long-term disability. However, for undetermined reasons, remyelination often fails in MS. Thus, enhancing remyelination is a therapeutic goal to prevent disability. Unfortunately, there is currently no such treatment available. In recent years, cell based therapy came into the focus of the different approaches to increase myelin regeneration [2]. Mesenchymal stem cells (MSC) are of particular interest since they secrete factors which are known to influence regeneration [3]–[5] and suppress immune cells [6], [7]. MSC are multipotent cells that can differentiate into different cell types such as osteocytes, adipocytes, and chondrocytes [8], [9]. Under *in vitro* conditions MSC can also generate neural-like and oligodendroglial-like cells [10]–[13]. It was also proposed that MSC might increase regeneration of oligodendrocytes and thus remyelination [14]. However, despite the potential to differentiate into different cell types many effects of MSC are thought to be mediated by creating an environment that forms the basis for the recruitment and stimulation of cells which are required for successful remyelination. These effects might be driven directly or might result from a modulation of the peripheral immune system [15]. To investigate such effects, different animal models and different ways of MSC application were tested by different groups [16]–[18]. Since direct injection of MSC into the lesion is difficult in MS patients, an intranasal (i.n.) or intravenous (i.v.) application might be a practical approach. In experimental autoimmune encephalomyelitis (EAE) i.v. application of MSC had a beneficial effect on the disease course [19]. The MSC were found in the lesions or near the lesions and in peripheral lymph nodes [15], [18], [20]. In healthy animals i.v. injected MSC were found predominantly in the lungs and only few MSC were found in the brain and spinal cord [19], [21]–[23].

Since the mechanisms how MSC enter the CNS are still not clear, we tested i.v. and i.n. applied murine and human MSC in the toxic cuprizone model of demyelination where the blood-brain-barrier (BBB) is intact and peripheral immune cells do not play a role [24]–[26].

## Materials and Methods

### Cell culture

Bone marrow aspiration from human donors was performed after consent of the ethics committee of Hannover Medical School. Written informed consent was obtained and all personal information including age and gender was rendered anonymous. For the present study, bone marrow was aspirated from the iliac crest during routine orthopedic procedures from one healthy donor. Aspirate was diluted with 3 volumes of PBS, filtered, and subjected to density gradient centrifugation with Biocoll (Biochrom AG, Berlin, Germany, ρ = 1.077 g/ml). The mononuclear cells were isolated from the interface, washed once in PBS, resuspended in medium and seeded into cell culture flasks. The medium contained DMEM (Biochrom AG, Berlin, Germany: FG0415) with 10% (vol/vol) FCS (Thermo Fisher Scientific “Hyclone”, Schwerte, Germany, not heat-inactivated), 20 mM 2-[4-(2-hydroxyethyl)piperazin-1-yl]ethanesulfonic acid (HEPES; Biochrom AG, Berlin, Germany), 100 U/ml penicillin, 100 µg/ml streptomycin (both from Biochrom AG, Berlin, Germany), 2 ng/ml human recombinant FGF-2 (Peprotech, Hamburg, Germany). The cells were cultured at 37°C, 5% CO_2_, 85% humidity. 24 hours after seeding, non-adherent hematopoietic cells were removed by washing. Further medium changes were performed every 3–4 days. Outgrowing colonies of plastic-adherent cells were detached with 0.025% trypsin-EDTA solution before reaching confluence and subcultured at a density of 2,000 to 5,000cells/cm^2^. Cells were used between passages 6 to 8 for the experiments. MSC characteristics were confirmed by flow cytometry of cell surface molecules as described previously [4].

Murine MSC isolated from bone marrow of C57BL/6 mice were purchased from Gibco. Cells were cultured in DMEM/F-12 medium with GlutaMAX™-I (Gibco, Karlsruhe, Germany) supplemented with 10% MSC-qualified fetal bovine serum (FBS, Gibco, Karlsruhe, Germany) and 100 U/ml penicillin, 100 mg/ml streptomycin (Sigma–Aldrich Chemical Co, Steinheim, Germany) for optimal growth and expansion. The subculturing and passaging cells was done after 1 time rinsing the surface of the cell layer with PBS without Ca^2+^ and Mg^2+^ (Gibco, Karlsruhe, Germany) and adding a sufficient volume of pre-warmed TrypLE™ Express to cover the cell layer (Gibco, Karlsruhe, Germany) to detach the cells.

The fibroblast NIH 3T3 cell line (generous gift from K. Wissel [27]) was maintained in Dulbecco's Modified Eagle's Medium (DMEM)-HAM's F-12 (1∶1) (Gibco, Karlsruhe, Germany) supplemented with 10% FBS (Biochrom, Berlin, Germany) and 1% penicillin/streptomycin (Sigma-Aldrich) in a 95/5% (vol/vol) atmosphere of air/CO_2_ at 37°C. The subculturing and passaging of cells was done as described before.

Immediately before application human MSC were characterised positive for CD73, CD105, and CD90 but negative for CD14b [28]. Mouse MSC were positive for CD29, CD44, Sca-1 and negative for CD45 [29] (figure S2).

MSC were labelled with PKH-26 according to the manufacturer's instructions (Sigma-Aldrich Inc., St. Louis, MO, USA) to track MSC after injection.

### Animals

*C57BL/6* male mice were obtained from Charles River (Sulzfeld, Germany). Animals underwent routine cage maintenance once a week and were microbiologically monitored according to Federation of European Laboratory Animal Science Associations recommendations [30]. Food and water were available *ad libitum*. All research and animal care procedures were approved (AZ 33.14-42502-04-11/0450) by the Review Board for the Care of Animal Subjects of the district government (Lower Saxony, Germany) and performed according to international guidelines on the use of laboratory animals.

### Induction of demyelination and MSC application

Demyelination was induced by feeding 8–10 week old mice with a diet containing 0.2% cuprizone (biscyclohexanone oxaldihydrazone, Sigma-Aldrich) mixed into a ground standard rodent chow. MSC were applied to the mice after 4 weeks of cuprizone feeding which is the known time point of strong microglial activation in the CNS [26]. Both murine or human MSC cells were applied as follows: for intranasal treatment animals received 2×60 U hyaluronidase (Sigma-Aldrich) in 6 µl *aqua dest.* per nostril, followed by 1×10^6^ MSC per animal in 24 µl PBS after 30 min. For intravenous administration 1×10^6^ MSC in PBS were injected into the tail vein. Control animals received cuprizone and NIH 3T3 fibroblasts as described above. Additional control groups were fed with normal food and received MSC i.n. and i.v. as described above or received cuprizone treatment without any cell application. Animals were sacrificed at week 4.5 and 5 of cuprizone treatment which is day 3.5 and 7 after cell injection. Blood was collected in EDTA for FACS analysis. Brains were removed and kept in PFA 4% in PBS overnight and afterwards in sucrose 30% in PBS for a minimum of 24 hours. Spinal cord of human MSC injected animals were also removed. Afterwards the tissue was kept frozen at −20°C in cryo embedding medium (O.C.T. ™ *Compound*, *Tissue Tek*®, Sakura Finetek Germany GmbH, Staufen, Germany). Spinal cord and brain were cut in 10 µm serial sections. Direct influence of light was avoided. To control for correct injection of the cells, lungs were removed and one half was used for immunohistochemistry. The other half was kept in ice cold PBS for flow cytometry (FACS) analysis.

### FACS analysis

MSC were characterised using a FACScalibur (BDscience, Franklin Lakes, New Jersey, USA) as previously described [31]. MSC were analysed for basic stem cell markers immediately before application. Human MSC were labelled with PE anti-human CD73, APC anti-human CD90, PE/Cy7 anti-human CD105 (BioLegend, San Diego, CA, USA) and FITC anti-human CD14 (eBioscience, San Diego, CA, USA). Mouse MSC were labelled with PE-anti-mouse CD29, PerCP/Cy5.5 anti-mouse CD44, FITC anti-mouse CD45, APC anti-mouse Sca-1 (BioLegend, San Diego, CA, USA). Additionally, MSC were stained with PE/Cy7 anti-human CD49d or FITC anti-mouse CD49d (both BioLegend, San Diego, CA, USA). Cells were also controlled for PKH-26 staining.

To control for the correct application of MSC and distribution in the mice, lungs were put through a 70 µm single cell strainer. Lung cells were crushed, dissociated and then diluted with PBS and centrifuged at 1500 g. Cells out of the supernatant were collected. Blood was treated with prewarmed erythrocyte lysis buffer. Both were analysed for PKH-26 positive cells.

### Immunohistochemistry

Immunohistochemistry was performed as previously described [32]. Briefly, frozen brain sections between bregma −0.94 mm and −1.34 mm (according to the mouse atlas by Paxinos and Franklin [33]) were stained for myelin, mature oligodendrocytes, astrocytes, and activated microglia.

Slides were thawed for 30 minutes and rehydrated in PBS, then sections were quenched with H_2_O_2_, blocked for 1 h in PBS containing 3% normal goat serum, 0.1% Triton X-100, and then incubated overnight with the primary antibody. The following antibodies were used: proteolipid protein (PLP, 1∶500, mouse monoclonal IgG2a, MorphoSys AbD GmbH, Düsseldorf, Germany) for myelin; Mac-3 (1∶500, rat IgG1, BD Pharmingen, Heidelberg, Germany) for activated microglia; glial fibrillary acidic protein (GFAP, 1∶200, polyclonal rabbit IgG, Dako Deutschland GmbH, Hamburg, Germany) for astrocytes; adenomatous polyposis coli (APC, 1∶200, mouse monoclonal IgG2b, Merck KGaA, Darmstadt, Germany) for mature oligodendrocytes. The day after washing, sections were further incubated with biotinylated anti-mouse IgG (H+L), anti-rat IgG (H+L), and anti-rabbit IgG (H+L) secondary antibodies (1∶500, Vector Laboratories, Burlingame, UK) for 1 h followed by peroxidase-coupled avidin–biotin complex (ABC Kit, Vector Laboratories, Inc., Burlingame, CA, USA). Reactivity was visualised with 3,3′-diaminobenzidine (DAB, Vector Laboratories, Inc., Burlingame, CA). For nucleus staining slides were counterstained using Mayer's hemalaun solution (Merck, Darmstadt, Germany). Stained slides were analysed by light microscopy (Olympus BX61, Hamburg, Germany).

### Quantification of glial cells

Quantification of glial cells was performed for oligodendrocytes (APC), astrocytes (GFAP), and activated microglia (Mac-3). Cells were considered as glial cells if they were positive by immunohistochemistry and hemalaun staining. The region of interest was the corpus callosum dorsal of the hippocampus. Positive cells were counted in the median part of the corpus callosum and in both lateral parts at a magnification of ×200 for astrocytes and oligodendrocytes and ×400 for microglia. The counted area was at least 0,12 mm^2^ for astrocytes and oligodendrocytes and 0,018 mm^2^ for microglia. Counted cells are presented as number of cells per mm^2^.

### Determination of de- and remyelination

Demyelination was identified by immunohistochemistry for the myelin marker PLP. The corpus callosum and the cortex were evaluated for the extent of myelination. A score from 3 in the corpus callosum and 4 in the cortex (physiological myelination) to 0 (complete myelin loss) was used. Scoring was performed by at least three blinded observers [34], [35]. Data are shown as average of all observers.

### Quantification of MSC

Per animal 10 serial frozen sections of *bulbus olfactorius*, rostral brain and spinal cord were stained with DAPI (Invitrogen, Carlsbad, CA) and screened for PKH-26 positive MSC by fluorescence microscopy. Cells were considered as positive if they showed red but no green fluorescence and if they were DAPI positive.

### Quantification of mRNA expression

To determine the mRNA levels of chemokines/growth factors expressed at week 4 (time point of acute demyelination and MSC application) a quantitative real-time PCR (qPCR) was performed. The corpus callosum was dissected from whole brains under a light microscope. Total RNA was extracted from the tissue using the RNeasy®Mini Kit (Qiagen, Germany) as previously described [36], [37]. cDNA was synthesized using the High Capacity cDNA Reverse Transcription Kit (Applied Biosystems, USA). Real-time quantitative PCR analysis was performed using the StepOne™ Real-Time PCR System and appropriate TaqMan probes (Applied Biosystems, USA). All primers were exon-spanning. The ΔΔCt method was used to determine differences in the expression of ccl2, cxcl-10, cxcl-12, VEGF, and HGF genes between cuprizone treated and control animals. Changes in the mRNA expression levels were calculated after normalization to *Hypoxanthin Phosphoribosyltransferase (HPRt)*.

### Cytotoxicity assay

Cellular viability was determined by incubation with AlamarBlue® (Resazurin, BioSource, Invitrogen, Oregon, USA), a non-toxic dye. For this purpose, murine MSC were seeded in a 96-well coated plate (Nunc, Steinheim, Germany), at a density of 5×10^4^ cells per well at 37°C in humidified air containing 5% CO_2_. As previously described, the cuprizone solution was prepared freshly, for each assay, by dissolving the cuprizone powder (Sigma-Aldrich) in 50% ethanol, at a 1 mM concentration [38]. This stock solution was further diluted in medium to obtain the required final concentration (50 µM, 100 µM, 500 µM). After 48 h incubation of murine MSC with different concentrations of cuprizone or with ethanol 50%, the cell viability was assessed by replacing the supernatant with DMEM containing 10% AlamarBlue®. Cells were further incubated for 3 h at 37°C. Optical density was measured at 570 nm using a spectrophotometer ELISA reader (Sunrise Basic Tecan, Crailsheim, Germany). As a negative control, AlamarBlue® was added to medium without cells. Triplicate measurements were averaged in four independent experiments.

### Statistical analysis

Statistical analysis for cellular reaction and demyelination as well as for MSC viability was performed using analysis of variance (ANOVA) followed by the Tukey's honestly significant difference test or Bonferonni post-hoc test for post hoc comparison if appropriate. If the data are not normally distributed Kruskal-Wallis-test was used followed by Dunn's multiple comparisons test. For the data of quantification of mRNA the Man Whitney test was used. All data are given as arithmetic means ± standard error of the mean (SEM). P values are given in the results while group comparisons derived from post hoc analysis are provided in the figures (*P<0.05; **P<0.01; ***P<0.001).

## Results

### No effect of human and mouse MSC on demyelination

**Figure 1:**
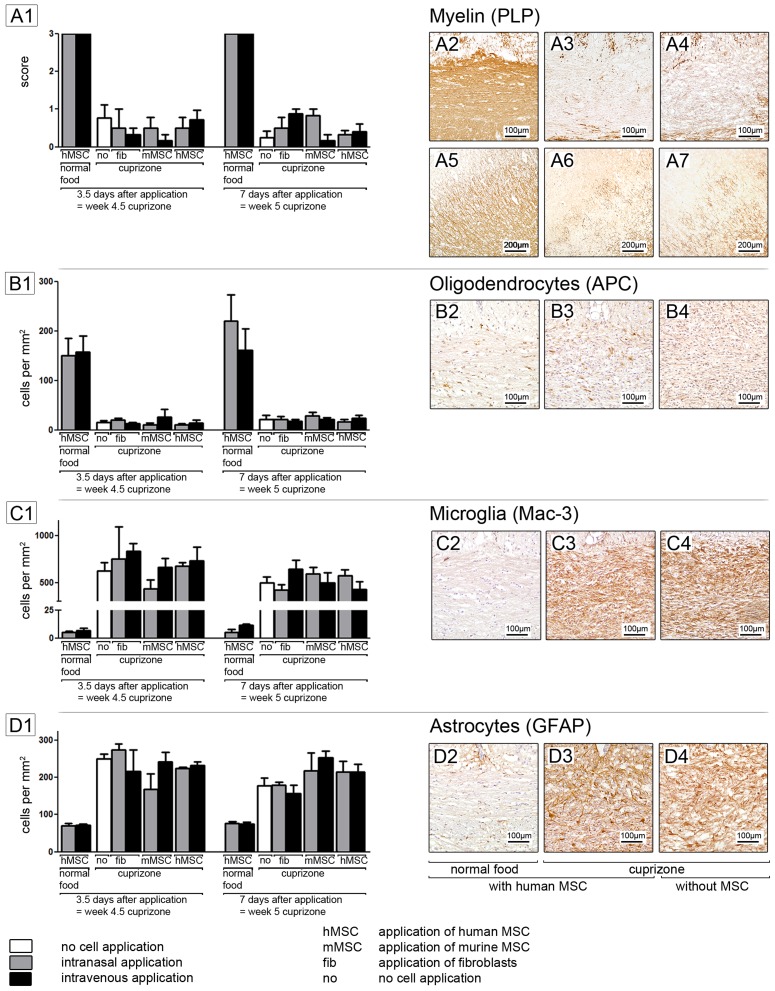
Immunohistochemical staining. MSC do not affect demyelination (**A1**) neither death of oligodendrocytes (**B1**). There is no impact of MSC on glial activation (**C1, D1**). Each bar represents the mean ± SEM. DAB immunohistochemical stainings of myelin (A2–A7), oligodendrocytes (B2–B4), microglia (C2–C4) and astrocytes (D2–D4) are shown exemplarily for the group of mice treated with cuprizone and human MSC (A3, A6, B3, C3, D3)and the control groups which received human MSC (A2, A5, B2, C2, D2) or cuprizone (A4, A7, B4, C4, D4) only.

To analyse the effects of human and mouse MSC on cuprizone induced demyelination two different administration routes were investigated. MSC were injected i.v. or were administered i.n. at 4 weeks after starting cuprizone feeding. The impact of MSC on cuprizone induced demyelination was analysed by immunohistochemical stainings for the myelin protein proteolipid protein (PLP). Mice fed with normal chow and treated i.n. and i.v. with human or murine MSC showed a physiological myelination in the corpus callosum with an intact structure. After 4.5 weeks of cuprizone feeding a significant loss of myelin was visible in all cuprizone groups as expected. Demyelination continued and was almost complete at week 5. No difference was found between mice treated with fibroblasts, human or mouse MSC using both administration routes or without MSC treatment (fig. 1 A1–A4).

In addition to the corpus callosum, the effect of human MSC on cortical demyelination was analysed, as human MSC were found in the cortical areas of the brain. Again, no difference in grey matter demyelination was found between cuprizone groups, showing that MSC did not protect from demyelination (fig. 1 A5–A7).

### Human and mouse MSC do not protect oligodendrocytes during cuprizone induced demyelination

In addition to demyelination, the effect of human and mouse MSC on oligodendrocytes was analysed by immunohistochemical staining for the marker APC. After feeding of cuprizone, APC positive oligodendrocytes decreased markedly to very low numbers. At week 4.5 no or only few oligodendrocytes were found in the corpus callosum in all cuprizone groups. In accordance to previous results [32], [34], [39], oligodendrocytes began to regenerate at week 5 as new APC positive cells were found in the corpus callosum. At both time points no difference was found between groups, suggesting that MSC have no impact on oligodendrocyte loss and regeneration (fig. 1 B1–B4).

### Human and mouse MSC have no impact on glial reactions

Microglial activation was investigated by Mac-3 staining while astrocytes were visualised using the marker GFAP. In all cuprizone groups strong microglia activation was found after 4.5 and 5 weeks of cuprizone treatment. Again, no difference was found between animals treated with human or mouse MSC with both administration routes (fig. 1 C1–C4).

The number of GFAP positive astrocytes was increased in all cuprizone groups at both time points. Again, no effects by MSC administration were found (fig. 1 D1–D4).

### Human MSC were found in low numbers in the CNS but not in the lesions

**Figure 2:**
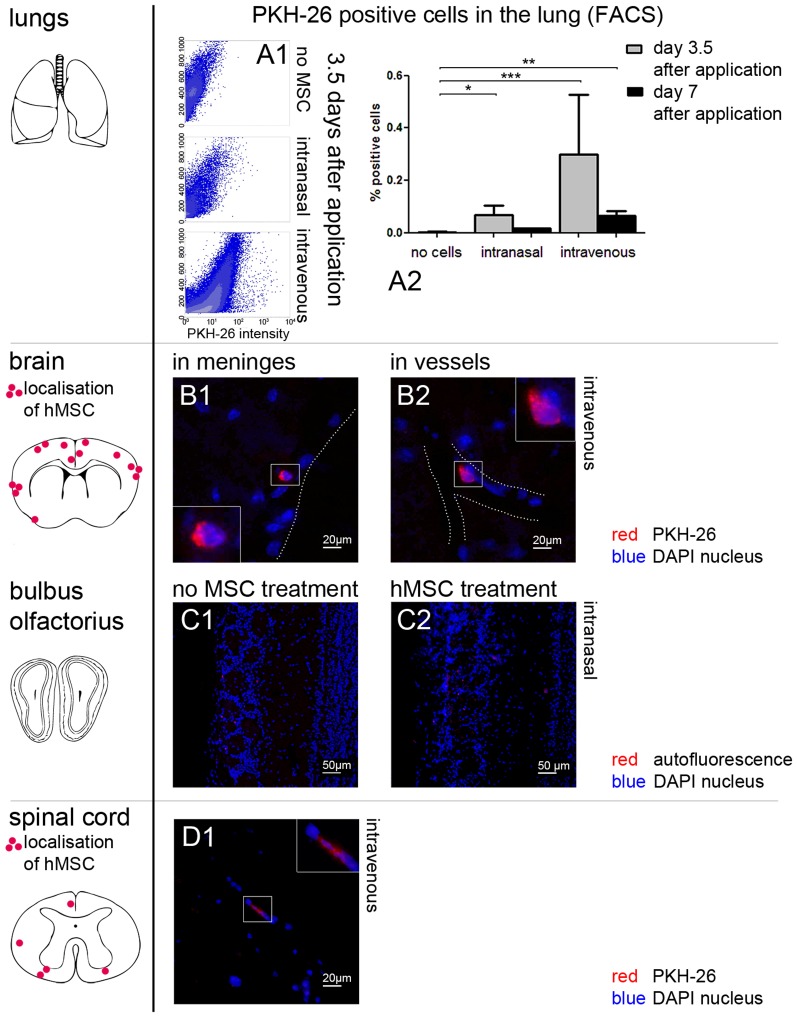
Cell tracking of human MSC with PKH-26. (**A1–A2**) After application of human MSC red fluorescent cells were found in the lungs of treated animals in the FACS analysis. (A2) Each bar represents the mean ± SEM. Significant differences between untreated and human MSC treated groups are indicated by asterisks (*P<0.05, **P<0.01, ***P<0.001). (**B1–B2**) The distribution of PKH-26 positive cells after i.v. injection of human MSC shows a hematogenic pattern in the brain. Human MSC (red) can be in or near the meninges (B1, here exemplarily day 7 after application) or seem to be attached to vessel walls (B2, here exemplarily day 3.5 after application). (**C1–C2**) In the *bulbus olfactorius* no red fluorescent cells were found in animals without cell treatment (C1) or after i.n. application of human MSC (C2). Here day 3.5 after application is shown exemplarily. Only some dim autofluorescence can be seen (red). (**D1**) PKH-26 positive cells (red) were found in very low numbers in the spinal cord after i.v. injection of human MSC (here exemplarily day 3.5 after application). For better visibility borders of parenchyma are illustrated with a dotted line (B1, B2). Nuclei were counterstained with DAPI (blue) (B1-D1).

Since no MSC effects were found on cuprizone induced demyelination and glial reactions, further analyses were performed in order to follow MSC after i.v. or i.n. administration. To assure the correct application the presence of MSC in the lungs and blood were investigated by FACS analysis and on frozen lung slides using fluorescent microscopy techniques. Both methods revealed that human and mouse MSC reached the lungs. As expected, the i.v. application led to higher numbers of MSC in the lungs compared to the i.n. in both normal chow and cuprizone fed mice (fig. 2 A1–A2). Unfortunately, neither human nor mouse MSC entered the demyelinated lesions.

However, after i.v. injection human MSC were found in low numbers in the brain and spinal cord of healthy and cuprizone fed mice (fig. 2 B1–B2, D1). In the spinal cord 0–2 human MSC per 10 serial slides were distributed mostly in the white matter (fig. 2 D1). In 10 serial sections of the brain only 0 to 3 human MSC could be detected. They showed a hematogenic distribution pattern as they were located in the peripheral grey matter or in the meninges but not in the areas of strong demyelination such as the corpus callosum. Some cells seemed to be attached to vessel walls (fig. 2 B1–B2). This result indicates that MSC do not cross the BBB and do not enter the damaged areas.

No human MSC were found in the CNS after i.n. injection (fig. 2 C1–C2).

Murine MSC could not be detected neither in the brain nor in the spinal cord indicating that murine MSC do not migrate into the CNS in mice with cuprizone- induced demyelination.

### Cuprizone is not toxic for murine MSC *in vitro*

To ensure that the absence of murine MSC in the CNS is not due to direct toxic effect of cuprizone, the MSC viability was investigated using the via Alamar blue assay. As shown in fig. S1 cuprizone is not toxic for murine MSC.

### Increased levels of mRNA expression of CCL-2, CXL-10 and HGF in the corpus callosum of cuprizone fed mice

**Figure 3:**
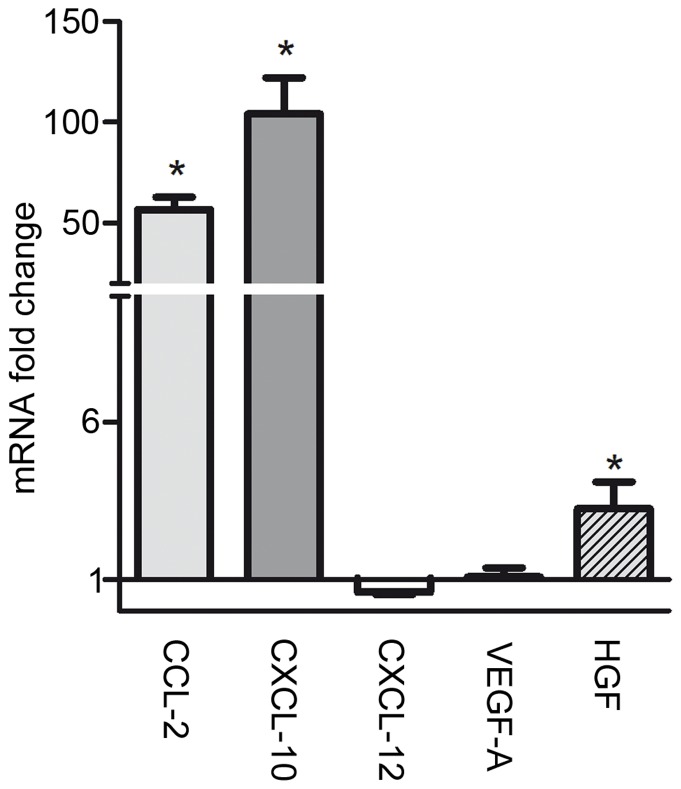
mRNA expression of chemokine and growth factors in the corpus callosum at week 4 of cuprizone feeding. The change of mRNA expression of CCL-2, CXCL-10, CXCL-12, VEGF-A, and HGF in the corpus callosum is shown at the time point of MSC application. Significant differences between untreated and cuprizone treated groups are indicated by asterisks (*P<0.05, **P<0.01, ***P<0.001).

The potential of the CNS to attract MSC into the lesion was quantified by PCR analysis of known chemotactic factors [40]–[42] on the mRNA level. During the time point of MSC application (week 4), a strong up-regulation of CCL-2 and CXCL-10 occurred, while HGF increased to a smaller extent. The expression of mRNA for CXCL-12 and VEGF-A was not changed (fig. 3).

### Human MSC but not mouse MSC show expression of the cell adhesion protein CD49d

In order to understand the differences between human and murine MSC we analysed the cells for the expression of the adhesion molecule CD49d. In FACS analyses about 38% of human MSC showed the expression of CD49d while mouse MSC were negative for this surface receptor (fig. S2).

## Discussion

Mesenchymal stem cells have been suggested to have regenerative effects in demyelinating diseases such as multiple sclerosis (reviewed by Uccelli [43]). After myelin damage, there can be a highly effective regenerative process, in which MSC might be activated and recruited to the lesion in order to increase the generation of new myelinating oligodendrocytes [44]. It has been discussed that such beneficial effects might be regulated by direct differentiation of MSC into myelinating cells [13]. However, recent evidence suggests that MSC rather might influence repair processes by providing the signal environment that forms the basis for the recruitment of oligodendrocyte progenitor cells and oligodendrocyte regeneration [3], [4], [45]. Still the mechanisms leading to myelin repair remain elusive.

We have therefore analysed the potential effects of human and murine bone marrow-derived MSC in a toxic model of cuprizone induced demyelination using two different application routes. In this animal model the BBB remains intact and it allows analysis of MSC effects without interference from the peripheral immune system. Using the cuprizone model we have shown here that neither human nor murine i.n. or i.v. applied MSC have an impact on demyelination or glial activation.

This lack of effect may depend on the lack of MSC in the lesions. No murine MSC were found in the brain and only a few human MSC which were applied i.v. were found inside the vessels of the brain, in the meninges, and in the spinal cord. We could not confirm the findings of others who showed high numbers of MSC [46] in the spinal cord even in naïve animals [19] or in the *bulbus olfactorius* [47]. This might be due to different evaluation methods with very strict criteria for the detection of PKH26 positive MSC in our experimental setting or a difference in age of the animals at the time of MSC administration. The analysis of chemotactic factors [40]–[42], [48], [49] in cuprizone induced demyelination revealed an increased expression of CCL-2, CXCL-10, and HGF in the corpus callosum suggesting a potential source of the CNS to attract MSC into the lesion. Since cuprizone is not toxic for MSC *in vitro* a direct toxic effect can be excluded.

The reason for the different findings between murine MSC and human MSC might be the lack of surface receptors such as CD49d in murine MSC which allow them to attach to vessel walls. It was already suggested that the presence of the cell adhesion receptor CD49d is an important factor for cells to migrate into the CNS [50]. Interestingly, in our experiments CD49d was found on human MSC, but could not be detected on murine MSC indicating that this receptor might indeed be of some relevance. However, this receptor seems not to be a main factor for cells to cross the BBB, since no human MSC were found in the lesions in the CNS parenchyma. Our results show that MSC are not able to enter demyelinating lesions in a toxic model with an intact BBB. At the time point of MSC application microglial and astroglial activation are at their peak and thus a strong inflammatory stimulus should be expected in order to attract MSC. Therefore it can be expected that if there is a signal that directs MSC into the lesion in this model it should be present at this time point. Differences in models (e.g. EAE) may explain this lack of migration of MSC into demyelinated lesions in the cuprizone model where there is an intact BBB and virtually no peripheral immune cells in the lesions.

In addition, our results indicate that neither human nor murine MSC created an oligoprotective or regenerative environment in the periphery that would be reflected in the CNS, since no beneficial effects were found on oligodendrocyte loss or their regeneration. The regenerative tendency seen in EAE [15] could point to the involvement of peripheral immune cells. It was demonstrated that most MSC applied via i.n. or i.v. routes become trapped in the lungs [21]. It was suggested that T cells need to reside in the lungs before being able to enter the CNS [51] and MSC might influence them in the priming process. In contrast to inflammatory models for MS, in the toxic cuprizone model the peripheral immune system does not play a role in CNS de- and remyelination [26]. Especially T cells are not affected, which seems to be an important target for MSC to affect CNS processes in inflammatory demyelination.

In conclusion, our data show that bone marrow-derived murine and human MSC do not affect the damage set by cuprizone and do not cross the intact BBB in this model.

## Supporting Information

Figure S1 **Viability of mMSC after cuprizone incubation** ***in vitro*** **.** Murine MSC were incubated with 50 µM, 100 µM, and 500 µM of cuprizone (CPZ) for 24 hours. The control groups were incubated with 50% ethanol or medium only. Cell viability is shown via Alamar blue assay.
(TIF)

Figure S2 **FACS analysis of MSC before application.** Human and murine MSC were analysed immediately before application. (**A**) Murine MSC show no unspecific staining (isotype control), are negative for CD45, but positive for the stem cell markers CD29, CD44 and Sca-1. (**B**) Human MSC show no unspecific staining (isotype control), are negative for CD14, but are positive for the stem cell markers CD73, CD105, CD90. Proper PKH-26 labelling is shown for both cell types. 38% of human MSC are positive for the cell adhesion protein CD49d while murine MSC are negative.
(TIF)
