# Supplementary figures and images for: Effects of Murine and Human Bone Marrow-Derived Mesenchymal Stem Cells on Cuprizone Induced Demyelination
Source: PLoS One. 2013 Jul 26;8(7):e69795. doi: 10.1371/journal.pone.0069795 (PMC3724887; doi:10.1371/journal.pone.0069795)

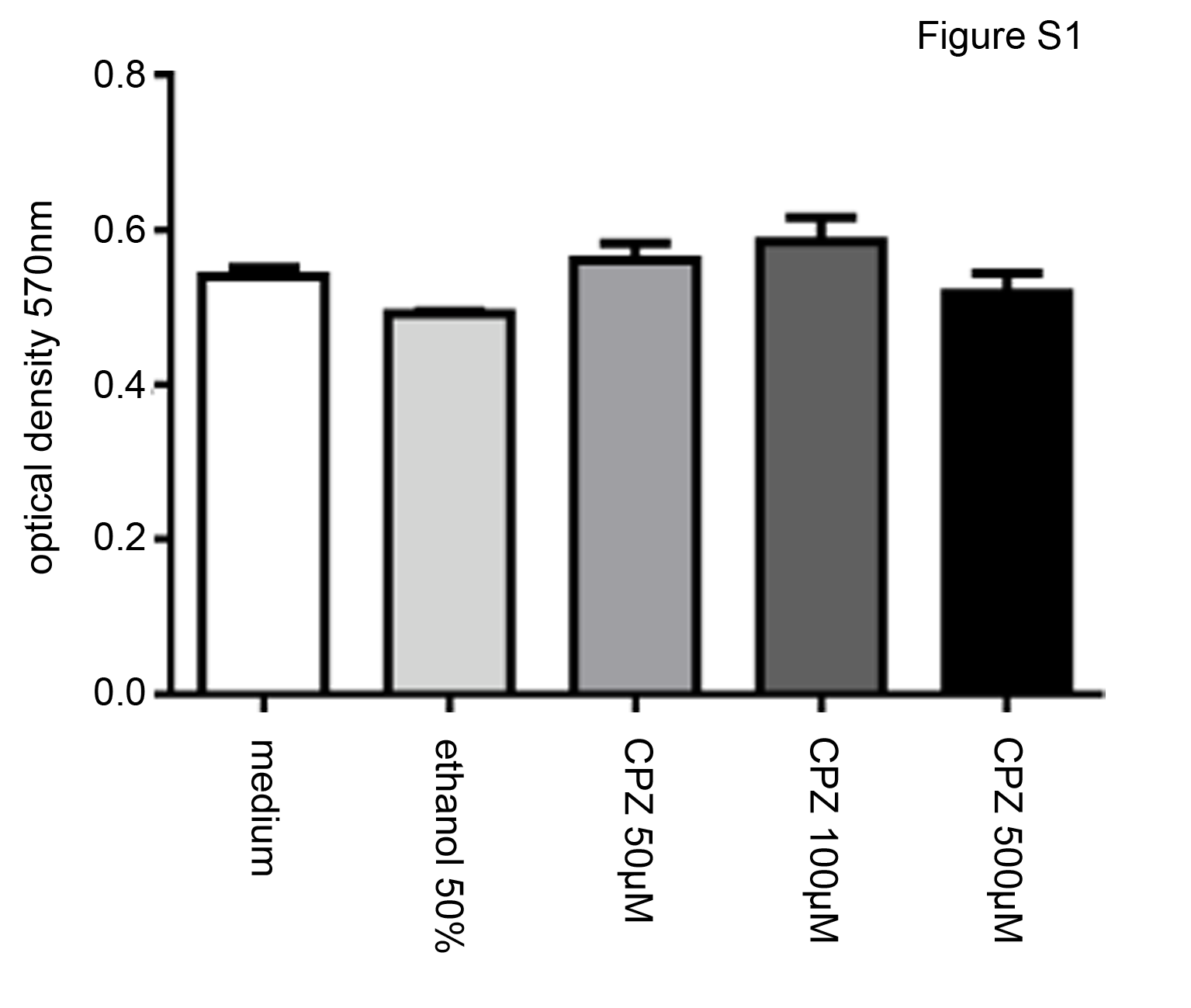

Supplement: Figure S1 — Viability of mMSC after cuprizone incubation in vitro . Murine MSC were incubated with 50 µM, 100 µM, and 500 µM of cuprizone (CPZ) for 24 hours. The control groups were incubated with 50% ethanol or medium only. Cell viability is shown via Alamar blue assay. (TIF) [file pone.0069795.s001.tif]

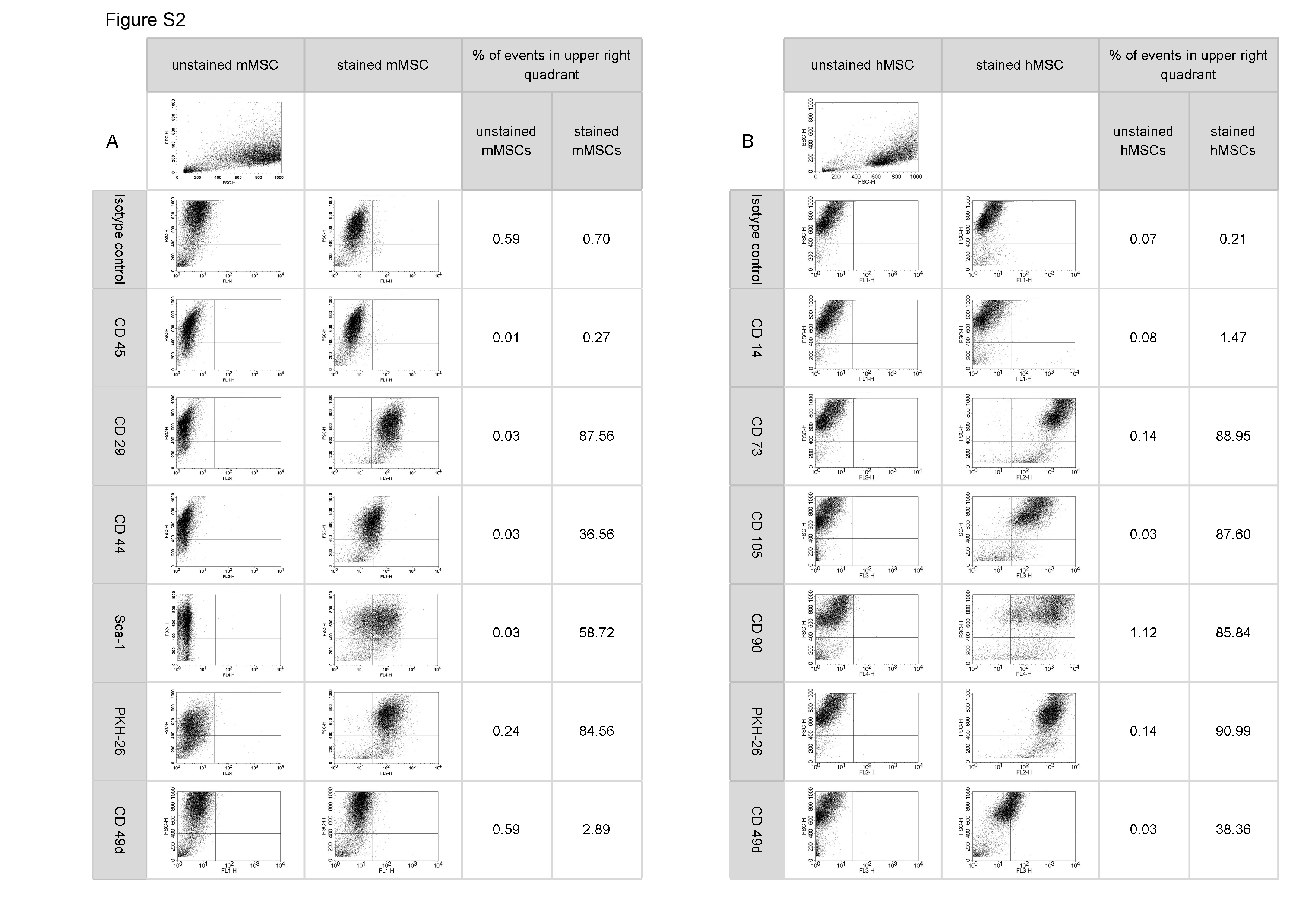

Supplement: Figure S2 — FACS analysis of MSC before application. Human and murine MSC were analysed immediately before application. (A) Murine MSC show no unspecific staining (isotype control), are negative for CD45, but positive for the stem cell markers CD29, CD44 and Sca-1. (B) Human MSC show no unspecific staining (isotype control), are negative for CD14, but are positive for the stem cell markers CD73, CD105, CD90. Proper PKH-26 labelling is shown for both cell types. 38% of human MSC are positive for the cell adhesion protein CD49d while murine MSC are negative. (TIF) [file pone.0069795.s002.tif]
